# Electrochemical Detection of Glyphosate in Surface Water Samples Based on Modified Screen-Printed Electrodes

**DOI:** 10.3390/nano14110948

**Published:** 2024-05-28

**Authors:** Elisabeta-Irina Geana, Corina Teodora Ciucure, Amalia Soare, Stanica Enache, Roxana Elena Ionete, Livia Alexandra Dinu

**Affiliations:** 1 National Research and Development Institute for Cryogenics and Isotopic Technologies—ICSI Rm. Valcea, 240050 Râmnicu Vâlcea, Romania; corina.ciucure@icsi.ro (C.T.C.); amalia.soare@icsi.ro (A.S.); stanica.enache@icsi.ro (S.E.); roxana.ionete@icsi.ro (R.E.I.); 2 National Institute for Research and Development in Microtechnologies (IMT Bucharest), 077190 Voluntari, Romania

**Keywords:** electrochemical sensor, glyphosate, molecularly imprinted polymer, pyrrole, gold nanoparticles, graphene

## Abstract

This study addresses the necessity to monitor the presence of glyphosate (Gly) in waters, highlighting the need for on-site detection of Gly by using electrochemical sensors in environmental and agricultural monitoring programs. Two approaches were employed: (1) modification with graphene decorated with gold nanoparticles (AuNPs-Gr) and dispersed in either dimethylformamide (DMF) or a solution containing Nafion and isopropanol (NAF), and (2) molecularly imprinted polymers (MIPs) based on polypyrrole (PPy) deposited on gold SPEs (AuSPE). Electrochemical characterization revealed that sensors made of AuNPs-Gr/SPCE exhibited enhanced conductivity, larger active area, and improved charge transfer kinetics compared to unmodified SPEs and SPEs modified with graphene alone. However, the indirect detection mechanism of Gly via complex formation with metallic cations in AuNPs-Gr-based sensors introduces complexities and compromises sensitivity and selectivity. In contrast, MIPPy/AuSPE sensors demonstrated superior performance, offering enhanced reliability and sensitivity for Gly analysis. The MIPPy/AuSPE sensor allowed the detection of Gly concentrations as low as 5 ng/L, with excellent selectivity and reproducibility. Moreover, testing in real surface water samples from the Olt River in Romania showed recovery rates ranging from 90% to 99%, highlighting the effectiveness of the detection method. Future perspectives include expanding the investigation to monitor Gly decomposition in aquatic environments over time, providing insights into the decomposition’s long-term effects on water quality and ecosystem health, and modifying regulatory measures and agricultural practices for mitigating its impact. This research contributes to the development of robust and reliable electrochemical sensors for on-site monitoring of Glyphosate in environmental and agricultural settings.

## 1. Introduction

In the global agriculture sector, the herbicide market is growing and competitive. Glyphosate, N-(phosphonomethyl)-glycine (Gly), an organophosphate pesticide that is commonly used worldwide, is a broad-spectrum, non-selective post-emergence herbicide that inhibits the shikimic acid pathway of plants [1]. It is widely used in agriculture to increase the tolerance of genetically modified crops, but also in horticulture, forestry, and in homes and gardens [2]. Commercial glyphosate formulations marketed for agricultural use commonly contain approximately 1–41% glyphosate.

Despite its retention in soil and its biodegradation into aminomethyl phosphonic acid (AMPA), glyphosate has been detected in soil and water long after application and sometimes far from the application site [3]. Glyphosate residues have the potential to contaminate surface waters and enter the food chain due to their high solubility in water, where glyphosate has a half-life ranging from a few days to 91 days [4]. Chronic exposure of Gly is associated with many human health hazards that include endocrine function disruption, colitis, diabetes, heart disease, and obesity.

Maximum residue limits (MRLs) allowed in drinking water differ from one regulatory agency to another. For example, MRL values of glyphosate in drinking water set by the EU and the US EPA are 0.10 and 700 μg/L, respectively [5,6]. Therefore, monitoring the content of glyphosate in drinking and surface waters, but also in fruits and vegetables, has become increasingly important.

Gly is considered a “difficult herbicide” in terms of trace analysis, due to the fact that it has a low molecular weight, high polarity, low volatility, thermal lability, good water solubility, and the lack of a chromophore or fluorophore in its molecular structure. These properties cause problems in Gly extraction, purification, and detection, with different derivatization methods being employed in several studies [7].

Traditional analytical methods for the quantitative determination of glyphosate have generally been based on chromatography, after a previous sample extraction step, such as liquid phase extraction or solid phase extraction [2]. Several methods have been developed for the detection of glyphosate, including HPLC [8,9], mass spectrometry [10,11], ion chromatography [12], gas chromatography [13], and capillary electrophoresis [14]. All these methods are time-consuming, require expensive equipment and qualified personnel, and cannot be employed for on-site measurements. For this purpose, there is an urgent need for a sensitive, selective, and responsive on-site monitoring methods [15,16]. Thus, electrochemical sensors have been intensively studied in the last few years as alternatives to traditional analytical methods [17,18,19].

The main challenge in the electrochemical detection of glyphosate is its poor electroactivity in the accessible potential window using conventional electrodes and media [20]. The most commonly used electrochemical techniques employed for the detection of glyphosate are amperometry, cyclic voltammetry (CV), square wave voltammetry (SWV), differential pulse voltammetry (DPV), all of which use different types of electrodes, such as carbon paste (CPE) [21], graphite pencil (PGE) [22], glassy carbon [23], gold [24,25], copper [26,27], carbon [21,22], mercury [28], and platinum [29]. In order to increase the sensitivity and selectivity for Gly detection in real samples, electrodes have been modified with different electroactive materials, such as graphene and graphene oxides [30,31,32], single and multi-walled carbon nanotubes (SWCNT, MWCNT) [23,33], copper or copper oxide (CuO) [26,34,35,36,37], copper phthalocyanine [38,39], and molecularly imprinted polymers (MIP) [40,41,42,43,44,45].

In recent years, graphene and metal nanoparticle-decorated graphene have garnered significant attention in the field of electrochemical sensing, particularly for their unique properties which enhance sensitivity and conductivity [46]. Graphene, a single layer of carbon atoms arranged in a hexagonal lattice, exhibits excellent electrical conductivity, a large surface area, and remarkable mechanical strength [47]. When decorated with metal nanoparticles, such as gold nanoparticles (AuNPs), the resulting nanocomposite combines the inherent properties of graphene with the catalytic activity and stability of the metal nanoparticles [46]. Gold nanoparticles, in particular, contribute to improved electron transfer kinetics and enhanced surface area, making them ideal candidates for modifying electrodes in electrochemical sensors [48]. The synergistic effects of graphene and gold nanoparticles offer an efficient platform for the development of highly sensitive and selective sensors for the detection of analytes such as glyphosate [49]. The electrochemical detection strategy for glyphosate utilizing gold-decorated graphene nanomaterial is based on the distinctive feature of glyphosate—the presence of the phosphonyl group. This characteristic endows glyphosate with the ability to form stable complexes with various metallic cations, including Fe^2+^ and Cu^2+^. This inherent property provides a promising avenue for indirect detection methodologies, leveraging the formation of stable complexes with metallic cations as a means to identify glyphosate in analytical systems [32,38,50]. By capitalizing on glyphosate’s affinity for metallic cations, the development of indirect detection approaches becomes not only feasible, but also offers the potential for heightened sensitivity and selectivity in glyphosate analysis.

On the other hand, molecularly imprinted polymers (MIPs), with a specific focus on polypyrrole (PPy), have emerged as promising materials for the development of electrochemical sensors [51]. MIPs are synthetic polymers designed to recognize and selectively bind to a target molecule with high affinity and specificity [52]. Polypyrrole, a conducting polymer, provides excellent electrical conductivity and stability, making it a suitable candidate for sensor applications [53,54]. The molecular imprinting process involves creating cavities in the polymer matrix that mimic the size, shape, and functional groups of the target molecule [55]. In the context of glyphosate detection, PPy-MIPs offer a tailored recognition interface for the herbicide, resulting in enhanced selectivity and sensitivity [42,56]. The incorporation of MIPs into the sensing platform contributes to the development of robust and reliable electrochemical sensors capable of discerning glyphosate in complex sample matrices with high precision.

In the pursuit of an efficient and practical electrochemical sensor for glyphosate detection, the current study strategically employed a screen-printed electrode as the electrochemical transducer. This choice was driven by the screen-printed electrode’s inherent characteristics, particularly, its miniaturization and integration features [57]. Recognizing the imperative need for portable devices capable of on-site measurements in diverse settings, the screen-printed electrode serves as an ideal platform for the development of the glyphosate sensor. Its miniaturized form facilitates ease of integration, ensuring the creation of a compact and portable electrochemical sensing device [58]. This deliberate selection aligns with the overarching goal of enhancing the practicality and applicability of glyphosate detection methods, emphasizing the study’s commitment to fostering advancements in on-the-go analytical solutions for environmental and agricultural monitoring.

The objective of this study was to develop indirect electrochemical sensors based on the use of screen-printed electrodes (SPEs) modified with different electroactive materials for the sensitive on-site detection of the herbicide glyphosate (Gly) in liquid samples. For this, screen-printed carbon electrodes (SPCEs) and screen-printed gold electrodes (AuSPE) were used as electrochemical transducers for the development of sensors that can be employed for the on-site detection of Gly. For the functionalization of the working detection electrode, two types of materials were used to develop electrochemical sensors: (1) graphene decorated with gold nanoparticles (AuNPs-Gr) showing high sensitivity as non-enzymatic material for sensor modification, and (2) molecularly imprinted polymer (MIP) with both high sensitivity and high selectivity towards the detection of Gly in synthetic water samples. The modified sensors were morphologically and electrochemically characterized, and their analytical sensor performances (linearity range and detection limit) were demonstrated in synthetic water solutions contaminated with Gly by using cyclic voltammetry (CV).

## 2. Materials and Methods

### 2.1. Reagents and Solutions

Dimethylformamide (DMF), nafion, and isopropanol (from Merck, Darmstadt, Germany) were used for graphene dispersion, while potassium ferrocyanide (K_4_[Fe(CN)_6_]), potassium chloride (KCl), and glyphosate (Gly) were procured from Sigma Aldrich (Steinheim, Germany). A 1-mM stock solution of K_4_[Fe(CN)_6_], employed as the redox probe, was prepared by dissolving 11.9 mg in 25 mL of 0.2 M KCl, serving as the supporting electrolyte. Gly was dissolved in water to create a 1000-mg/L stock solution. For lower concentrations of Gly, the serial dilution method was utilized, performed in 0.1 mol L^−1^ KCl or in 1.0 mmol L^−1^ K_4_[Fe(CN)_6_] (in 0.1 mol L^−1^ KCl) supporting electrolytes. The graphene decorated with gold nanoparticles (AuNPS-Gr) used for SPE modification was previously synthesized by using an environmentally friendly method, namely, microwave-assisted hydrothermal synthesis, starting from graphene oxide [59]. The AuNPs-Gr was characterized by structural, spectroscopic, chemical [59], and morphological [60] methods.

### 2.2. Electrochemical Apparatus and the Functionalization Procedures

The electrochemical measurements were conducted using an AUTOLAB 302N potentiostat/galvanostat. Carbon screen-printed electrodes (SPCE) from DropSens (model DRP 110) were employed as transducers for sensors modified with graphene and consisted of the following: the working electrode (WE)—carbon ink, the counter electrode (CE)—carbon ink, and the reference electrode (RE)—Ag ink pseudo electrode. Additionally, gold screen-printed electrodes (AuSPE) from DropSens (model DRP 250BT) were utilized for sensors modified with molecularly imprinted polymers (MIP) with the following configuration: WE—gold ink, CE—gold ink, and RE—Ag ink pseudo electrode. The electrochemical measurements were conducted in a 10-mL electrochemical cell and a small-volume electrochemical cell for volumes ranging from 100 to 1000 μL.

Before conducting measurements, the electrodes were activated in a saline solution (0.2 M KCl) through 50 scan cycles at a scan rate of 50 mV/s to obtain stable voltammetric responses. Typically, for each solution to be measured, two stabilization cycles were performed in the respective sample, followed by the actual measurements (*n* = 3).

For the first designs of the sensors, AuNPs-Gr was either dispersed in DMF, or in water containing nafion and isopropanol solution (NAF). These modified sensors have been electrochemically characterized using cyclic voltammetry (CV) and electrochemical impedance spectroscopy (EIS), showing a larger active area (AA) and a low charge transfer resistance (Rct) for the sensors modified with AuNPs-Gr/SPCE, compared to the Gr/SPCE and bare SPCE.

**Figure 1:**
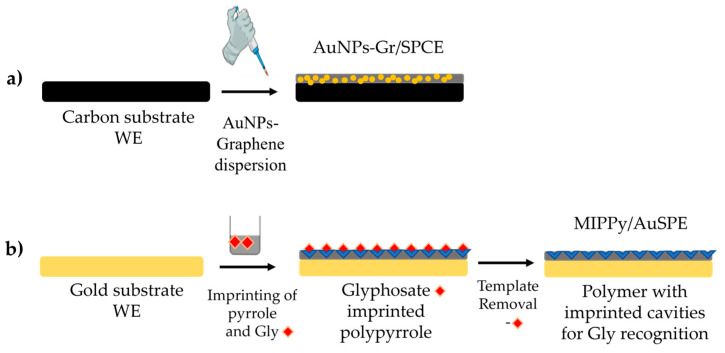
The schematic representation of the functionalization step with (**a**) AuNPs-rGO and (**b**) MIPPy of the working electrode (WE).

The immobilization of graphene-based nanomaterials on the working electrode surface (Figure 1a) was achieved by depositing a 10-μL dispersion obtained either by mixing AuNPs-decorated Graphene in dimethylformamide (DMF) or an aqueous solution containing Nafion and isopropanol. Typically, for each sample solution, two stabilization cycles were performed in the respective sample, followed by the actual measurements (*n* = 3).

The modification of SPE electrodes to obtain molecularly imprinted polymers (MIPs) was accomplished through the electro-polymerization of the 0.1-mol L^−1^ KCl supporting electrolyte containing 37.0 mM Py and 7.4 mM Gly on the working electrode surfaces made of Au. This process occurred within the potential range of −1.0 to +1.0 V at a scan rate of 100 mV/s for five cycles. Following electro-polymerization, the incorporated Gly molecules were extracted from the Ppy membrane using a superoxidation method involving CV scanning in the potential range of −1.3 to +1.2 V at a scan rate of 100 mV/s in a 0.1-M NaOH solution over 20 cycles. This resulted in an MIPPy electrode with specific cavities for Gly. For comparison, a non-imprinted SPE electrode (NIPPy) was prepared under the same experimental conditions, except that the Gly template was omitted from the pre-polymer solution. The protocol for obtaining the MIPPy electrodes is schematically depicted in Figure 1b.

## 3. Results

### 3.1. Structural and Chemical Characterization of AuNPS-Gr Nanohybrid Material

The AuNPS-Gr nanohybrid material was previously characterized by structural, spectroscopic, and chemical investigations [59]. Thus, in order to investigate the structure and characterize the porosity of doped graphene, the nitrogen adsorption–desorption isotherms were performed using the Brunauer–Emmett–Teller (BET) method. The BET surface area corresponding to the AuNPS-Gr was estimated to be 244.3 m^2^·g^−1^. Chemical composition of the AuNPS-Gr was established in terms of elemental composition (55.10% C, 1.01% H, 0.37% N, 0.47% S and 10.29% O) and Au content (15.71%) determined by atomic absorption spectroscopy. The FT-IR spectra of AuNPS-Gr exhibit several characteristic peaks. The C–O-stretching vibration, indicating the presence of remaining oxygen functionalities, appears at 1250 cm^−1^. The C=C-stretching vibrations, corresponding to the carbon atoms in the graphene layer, are observed at 1590 cm^−1^. The C=O-stretching vibration, initially present at 1723 cm^−1^, is significantly weakened following the reduction of graphene. Furthermore, the broad signals around ~3450 cm^−1^ are attributed to the O–H stretching mode of intercalated water [59]. The SEM micrographs showed ultrathin graphene oxide (GO) sheets hosting gold nanoparticles (AuNPs) sized between 5 and 100 nm. These graphene sheets exhibit a random porous structure with folds and wrinkles, forming an interconnected network crucial for electronic transport. The porous architecture ensures a uniform distribution of AuNPs, as observed in micrographs obtained with the HA-BSE detector [60].

### 3.2. Morphological Characterization of the Molecularly Imprinted Polypyrrole

The modification of the AuSPE electrode surface through the formation of specific recognition cavities for Gly was pursued through scanning electron microscopy (SEM) and atomic force microscopy (AFM). The investigation of the morphology of the polymeric films formed in the absence and presence of the Gly template highlighted the alteration of the Au electrode morphology (Figure S1a) upon substrate modification through electro-polymerization in the presence of pyrrole. The MIP film is compact before template removal (Figure S1b), while after template removal, some discontinuities in the polymeric film are observed (Figure S1c), resembling cavities.

The topography of the specimens was also investigated using AFM microscopy in AC mode (tapping) (Witec, Ulm, Germany, alpha300 RAS+) on surfaces of up to 25 × 25 μm^2^. The results indicate slight modifications following the deposition of polymer layers. For example, from the image analysis for the Au substrate in Figure S2, the roughness parameters SA and SQ are in the ranges of hundreds of nanometers with a negative skewness value (SSK), denoting material grains that appear as protrusions with a height distribution relative to the mean plane shifted to the right of the mean value (i.e., in the histogram of local maxima). Gold electrodes coated with the polymer exhibit similar roughness values, with the difference being that the presence of Gly shifts the histogram of local heights to higher values (i.e., SSK = −0.445) compared to the specimen where Gly is removed (i.e., SSK = −0.244).

### 3.3. Electrochemical Characterization of the Modified SPEs

#### 3.3.1. Electrochemical Characterization of the AuNPs-Gr/SPCE

**Figure 2:**
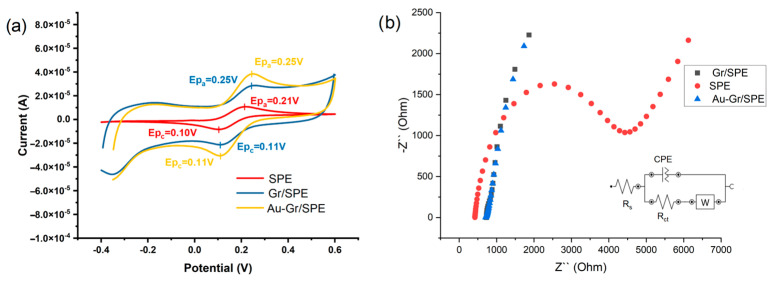
(**a**) Cyclic voltammograms (CVs) recorded in a solution of 1.0 mmol L^−^^1^ K_4_[Fe(CN)_6_] (in 0.1 mol L^−^^1^ KCl) using the unmodified SPE (red), Gr/SPE (blue), and Au-Gr/SCE (yellow); (**b**) Electric impedance spectra (EIS) of the microsensors based on SPE (red), Gr/SPE (black), and AuGr/SCE (blue) recorded in 1.0 mmol L^−^^1^ K_4_[Fe(CN)_6_] (in 0.1 mol L^−^^1^ KCl).

The modified sensors were electrochemically characterized using cyclic voltammetry (CV) and electrochemical impedance spectroscopy (EIS), demonstrating a larger active area (AA) and a low charge transfer resistance (Rct) for sensors modified with AuNPs-Gr/SPCE compared to Gr/SPCE and unmodified SPCE. To perform the electrochemical characterization of SPCE electrodes modified with Au-doped graphene, cyclic voltammograms were recorded in a 1.0-mmol L^−^^1^ K_4_[Fe(CN)_6_] (in 0.1 mol L^−^^1^ KCl supporting electrolyte) (Figure 2a). For EIS measurements, the frequency range was set between 0.1 and 10^5^ Hz, and the applied potential was the apparent potential (E°) of the redox couple. Inset: Equivalent circuit of the electrochemical interface used for fitting the results obtained through impedance measurements: R_s_—solution resistance; W—Warburg diffusion constant; R_ct_—charge transfer resistance, and CPE—constant phase element (Figure 2b).

In the CVs (Figure 2a) recorded in a 1.0-mmol L^−1^ K_4_[Fe(CN)_6_] solution with a 0.1-mol L^−1^ KCl supporting electrolyte, the observed increase in the sensor’s conductivity upon the deposition of Au-doped graphene on the SPCE surface is indicative of enhanced charge transfer kinetics. This rise in conductivity reflects the efficient modification of the electrode surface, allowing for improved electron transfer during redox processes. The higher conductivity is a crucial factor contributing to the overall electrochemical performance of the sensor, leading to a more pronounced and distinct electrochemical signal.

The EIS measurements further support the enhanced performance of the AuNPs-Gr/SPCE-modified sensor. The impedance spectra, as depicted in Figure 2b, provide detailed information about the electrochemical processes occurring at the electrode–electrolyte interface. The distinct features in the impedance spectra indicate changes in the charge transfer resistance (R_ct_) and, consequently, the kinetics of the electrochemical reactions. The AuNPs-Gr/SPCE-modified sensor exhibits lower charge transfer resistance compared to Gr/SPCE and unmodified SPCE, indicating more facile electron transfer and superior electrocatalytic activity.

The combination of CV and EIS data highlights the synergistic effect of gold nanoparticles and graphene modification on the SPCE, resulting in a sensor with enhanced conductivity, larger active area, and improved charge transfer kinetics. These improvements are crucial for the sensitive and selective detection of target analytes, making the AuNPs-Gr/SPCE-modified sensor a promising candidate for electrochemical sensing applications in the indirect detection of Gly.

#### 3.3.2. Electrochemical Characterization of the MIPPy/AuSPE

**Figure 3:**
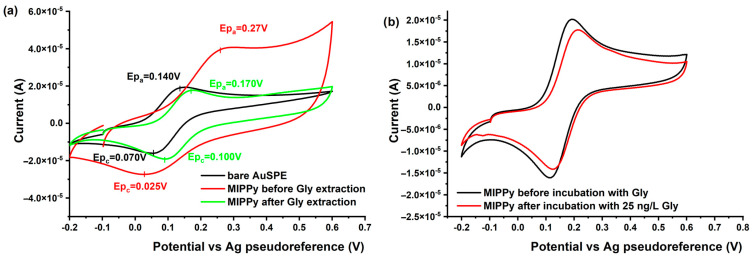
(**a**) Electrochemical characterization of the Au sensor (SPE) modified with molecularly imprinted polypyrrole (MIP-PPy) in solutions containing 1 mM K_3_FeCN_6_, and (**b**) Cyclic voltammograms recorded using the SPE sensor modified with MIPPy, in the absence of Gly (blue) and in the presence of Gly (orange).

To confirm that Gly molecules were incorporated into the imprinted PPy membrane, cyclic voltammetry (CV) measurements were conducted for various imprinted electrodes in a supporting electrolyte containing 1.0 mmol L^−1^ K_4_[Fe(CN)_6_] (in 0.1 mol L^−1^ KCl) (Figure 3a). The observed decrease in current intensity with the increasing concentration of Gly provides evidence that Gly molecules are effectively blocking the electrochemical active sites of the MIPPy sensor. This phenomenon leads to an amplified separation between the oxidation and reduction peaks (ΔEp), as illustrated in Figure 3b. The larger ΔEp suggests a hindrance in the electrochemical processes, indicative of specific interactions between Gly and the imprinted cavities within the PPy membrane. These results further confirm the successful molecular imprinting of Gly in the polymer matrix, highlighting the potential of the MIPPy sensor for the selective detection of Gly.

### 3.4. Voltammetric Detection of Gly Using Modified SPE

#### 3.4.1. Voltammetric Detection of Gly Using AuNPs-Gr/SPCE

**Figure 4:**
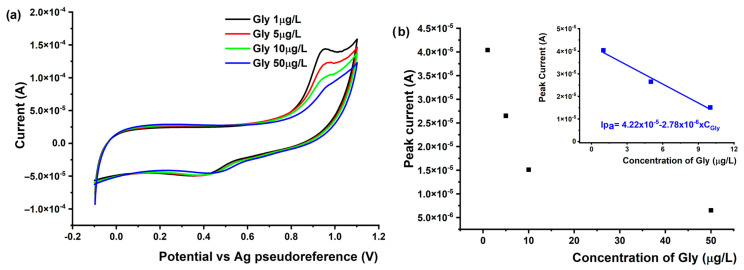
(**a**) Cyclic voltammograms (CVs) recorded using the AuNPs-Gr(DMF)/SPCE sensor in a phosphate buffer containing different concentrations of Glyphosate (Gly) (1–50 μg/L); and the (**b**) calibration curve in solutions containing different concentrations of Glyphosate (Gly) (1–50 μg/L).

The CV measurements conducted in the potential range of −0.2–1.2 for the SPCE sensor modified with a graphene–gold nanocomposite dispersed in DMF (AuNPs-Gr(DMF)/SPCE) in the presence of various concentrations of Glyphosate (Gly) in phosphate buffer revealed an electrochemical signal attributed to the oxidation–reduction of gold nanoparticles on the graphene surface. Given that glyphosate forms complexes with metal ions, the increase in glyphosate concentration is observed to decrease the signal intensity, allowing an indirect determination of glyphosate (Figure 4a). Gly, a phosphonate compound, contains a phosphorus atom surrounded by oxygen atoms, making it capable of forming coordination complexes with metal ions. Gly typically exists in its anionic form in aqueous solutions, carrying a net negative charge due to the presence of the carboxyl and phosphonate groups, among others. On the other hand, gold cations, such as Au(III), are positively charged. This difference in charge between glyphosate and gold cations suggests that their interaction would likely involve electrostatic forces. The recorded signal using the AuNPs-Gr(DMF)/SPCE sensor decreases with repeated measurements, highlighting the poor reproducibility of the AuNPs-Gr(DMF)/SPCE sensor. Glyphosate adsorbs onto the electrode surface, thereby hindering charge transfer, rendering the sensor suitable for a single measurement (considered for single-use applications) (Figure 4). This linear relationship between Glyphosate (Gly) concentration and the response of the AuNPs-Gr(DMF)/SPCE electrode, depicted in Figure 4b, signifies the sensor’s ability to provide a proportional and reliable signal across a concentration range of 1–10 μg/L. The analytical performance metrics derived from this study are summarized in Table 1. These performance indicators, including sensitivity, detection limit, and linear range, are crucial in assessing the effectiveness of the AuNPs-Gr(DMF)/SPCE sensor for detecting Glyphosate in aqueous solutions. The obtained results underscore the sensor’s capability to offer accurate and sensitive measurements, demonstrating its potential utility in environmental monitoring or other applications where precise detection of Glyphosate concentrations is essential.

**Figure 5:**
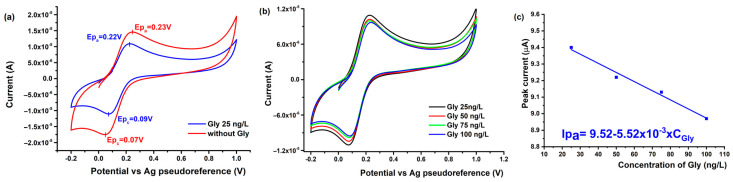
(**a**) CVs recorded using the AuNPs-Gr(NAF)/SPCE sensor in 1 mM K_3_FeCN_6_, in the presence of Gly (blue), and in the absence of Gly (orange); (**b**) CVs recorded using the AuNPs-Gr(NAF)/SPCE sensor in solutions containing various concentrations of Gly (1–100 ng/L); and (**c**) Calibration curve of the AuNPs-Gr(NAF)/SPCE sensor in solutions containing various concentrations of Gly (25–100 ng/L).

The CV measurements conducted in solutions containing Gly prepared in 1 mM K_3_FeCN_6_ with 0.1 M KCl supporting electrolyte revealed an electrochemical signal attributed to the Fe redox couple. The current intensity exhibited a decrease with the increasing concentration of Gly, indicating that Gly interferes with the active electrochemical centers of the AuGr-Nafion/SPCE sensor, resulting in a larger peak separation (ΔE_p_) (Figure 5a). The analytical performance of the AuNPs-Gr(NAF)/SPCE sensor was evaluated by measuring its response when incubated with various concentrations of Gly under optimized conditions (Figure 5b).

These observations highlight the sensor’s ability to detect Gly through the modulation of the Fe redox signal, and the increased ΔE_p_ serves as a quantitative indicator of the analyte’s presence. The analytical characteristics of the AuNPs-Gr(NAF)/SPCE sensor were further scrutinized, providing valuable insights into its sensitivity and selectivity when exposed to different Gly concentrations. These findings contribute to a comprehensive understanding of the sensor’s performance and its potential applications in the accurate and reliable detection of Gly in aqueous solutions.

It has been observed that there is a linear relationship between the concentration of Glyphosate (Gly) and the response of the AuGr-Nafion/SPCE sensor from 25 to 100 ng/L, as depicted in Figure 5c. The analytical performance metrics obtained from this linear correlation are comprehensively presented in Table 1. This linear correlation signifies the sensor’s ability to provide a consistent and proportional response across the specified concentration range of Glyphosate. The quantitative data presented in Table 1 further detail the sensor’s sensitivity, limit of detection, and other key analytical parameters, shedding light on its performance characteristics under various Gly concentrations.

#### 3.4.2. Voltammetric Detection of Gly Using MIPPy/AuSPE

**Figure 6:**
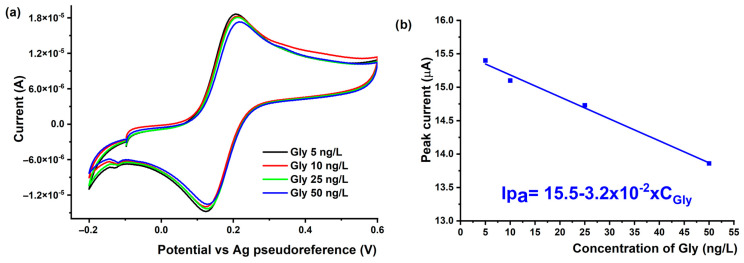
(**a**) CVs recorded using the MIPPy/AuSPE in solutions containing different concentrations of Glyphosate (5–50 ppt) and (**b**) the calibration curve.

The analytical performance characteristics of the MIPPy sensor were tested by measuring its response when incubated with various concentrations of Gly under optimized conditions. Thus, there was a linear relationship between the concentration of Gly and the response of the MIPPy electrode from 5 to 50 ng/L, as shown in Figure 6.

It is observed that at concentrations higher than 50 ng/L, the performance of the MIPPy/AuSPE sensor decreases, indicating a decrease in the number of electrochemically active centers on the electrode surface. Consequently, the sensor is capable of measuring very low concentrations of Gly. The limit-of-detection (LOD) for Glyphosate provided by the MIPPy/AuSPE electrode was calculated using the equation LOD = 3 Sb/m, where Sb is the standard deviation of the blank solution, and m is the slope of the calibration curve. The analytical performance of the MIPPy/AuSPE sensor for Gly (linear concentration range, correlation coefficient (r), limit-of-detection (LOD), limit-of-quantification (LOQ), and sensitivity) are presented in Table 2.

As a comparison, MIPPy’s excellent electrical conductivity and stability, inherent to polypyrrole, contribute to reliable sensor performance and lower LOD. In contrast, although AuNPs-Gr combines the conductivity of graphene with the catalytic activity of gold nanoparticles, its detection mechanism relies on indirect methods via the formation complexes with metallic cations. This indirect approach may introduce complexities in real-world applications and compromise sensitivity and selectivity. Therefore, MIPPy emerges as a superior choice for Gly analysis, offering enhanced performance and reliability for on-site detection in diverse environmental and agricultural matrices, and for these reasons, we continued our studies using the MIPPy/AuSPE to prove its analytical applicability.

Both approaches allowed the measurement of Gly concentrations below the maximum residual limit allowed in the European Union (0.1 μg/L for individual pesticides). Both showed similar or higher performances compared to other sensors used for Gly determination in aqueous samples by using different nanomaterials and MIPs for sensor modification (Table 3).

#### 3.4.3. Selectivity Studies and Analytical Application of Gly Using MIPPy/AuSPE

The selectivity studies of the developed MIPPy-AuE sensor demonstrated its robustness in distinguishing Gly from potential interferent species commonly found in environmental samples. Analyzing Gly at a concentration of 25 ng/L, the sensor exhibited a retained signal ranging from 93.3% to 104%, indicating its ability to accurately detect Gly in the presence of interferents. The selected interferent species in concentrations of 1:1, 1:10, and 1:50, including glufosinate ammonium (GLU), chlorpyrifos (CLP), and phosmet (PHO), were effectively discriminated from Gly, with negligible interference observed. Additionally, other potential pollutant molecules, such as tannic acid (TA), catechol (CAT), and bisphenol A (BPA), were considered and showed minimal impact on the sensor’s response (Figure S3a). The low relative standard deviation (RSD) values between 0.65% and 3.35% further underscore the sensor’s reproducibility and reliability in selective glyphosate detection. These findings highlight the MIPPy-AuSPE sensor’s promising potential for accurate and selective on-site monitoring of glyphosate in surface water samples, crucial for ensuring the quality of drinking water and the health of the population.

To evaluate the reproducibility, repeatability, and stability of the MIPPy-AuSPE for Gly detection, cyclic voltammetry (CV) measurements were conducted in a buffer solution containing 25 ng/L of Gly. This concentration was chosen to mimic real-world scenarios and assess the sensor’s performance under environmentally relevant conditions. The CV measurements were carried out according to standardized protocols to ensure consistency and accuracy in data acquisition. Reproducibility was assessed by comparing the peak current obtained from five MIPPy-AuSPE sensors under identical experimental conditions, demonstrating the sensor’s ability to consistently generate reproducible results across different sensor units (Figure S3b). Repeatability was evaluated by conducting successive scans using the same MIPPy-AuSPE sensor, illustrating the sensor’s capacity to yield consistent measurements over repeated trials (five replicates) (Figure S3c). Additionally, stability testing involved prolonged storage of the MIPPy-AuSPE sensor by assessing its long-term performance after 10, 20, 30, and 45 days. After 45 days in storage, the decrease in signal was significant, and we can conclude that a maximum duration of 30 days is a reliable storage time (Figure S3d). These comprehensive assessments provide insights into the robustness and reliability of the MIPPy-AuE sensor for Gly detection.

In our study, water samples were collected from the Olt River in Valcea County, Romania, in september 2022, and Gly was not detected in these samples. Therefore, the surface water samples were spiked with 10 and 30 ng/L to simulate different levels of contamination commonly found in agricultural runoff samples. The recovery rates observed, ranging from 90% to 99%, indicate the effectiveness of our detection method in accurately quantifying Gly levels in real-world water samples. However, considering the inherent degradation of Gly over time, it becomes imperative to collect water samples immediately after the application in agriculture. This timing ensures that the samples accurately reflect Gly concentrations shortly after application, providing crucial insights into the immediate environmental impact of Gly usage.

## 4. Conclusions

The electrochemical sensors developed in this study exhibit significant analytical performance for measuring Glyphosate (Gly) content in aqueous samples. Thus, the AuNPs-Gr(NAF)/SPCE sensor allowed the measurement of Gly concentrations within a concentration range of 20–100 ng/L, and a detection limit of 0.3 ng/L. The AuNPs-Gr(DMF)/SPCE sensor allow the measurement of Gly concentrations, within a concentration range between 1–50 μg/L, and a detection limit of 0.3 μg/L, indicating better performances when the SPEs were modified with AuNPs-Gr dispersed in NAF, compared with DMF.

In order to demonstrate the selectivity for Gly detection, molecularly imprinted polymers, which are robust molecular recognition materials with antibody-like capabilities for binding and discriminating between molecules, were tested for Gly detection in aqueous samples. The MIPPy/AuSPE sensor allowed the measurement of Gly concentrations within a concentration range of 5–50 ng/L, and a detection limit of 0.3 ng/L. MIP-PPy/AuSPE demonstrate better analytical performance for Gly detection in real surface water samples, which is useful for surface water quality monitoring studies affected by the intensive use of Gly in agriculture.

## 5. Future Perspectives

Our research will continue to assess the selectivity of the proposed sensor for determining Gly content in the presence of other environmental contaminants that may interfere with Gly detection (e.g., Zn^2+^, Cd^2+^, Ca^2+^, Mg^2+^, Na^+^, NH^4+^, Br^−^, NO^3−^, SO4^2−^, PO4^3−^). As a future perspective, it is essential to expand our investigation to include monitoring water samples at various time intervals after exposure to Gly. This longitudinal approach will enable us to understand the dynamics of Gly decomposition in aquatic environments and its potential long-term effects on water quality and ecosystem health. By gaining a comprehensive understanding of Gly’s behavior in natural water bodies, we can better inform regulatory measures and agricultural practices to mitigate its impact on the environment and human health.

## Figures and Tables

**Table 1:** Analytical parameters obtained for the determination of Gly using both AuGr-DMF/SPCE and AuGr-Nafion/SPCE sensors.

Analytical Parameter	AuNPs-Gr(DMF)/SPCE	AuNPs-Gr(NAF)/SPCE
Linear concentration range	1.0–50.0 μg/L	20.0–100.0 ng/L
Detection limit	0.3 μg/L	6.6 ng/L
Quantification limit	1.0 μg/L	1.0 ng/L
Sensitivity	−2.78 μA/μg/L	−5.52 × 10^−3^ μA/ng/L
Correlation coefficient (r)	0.9854	0.9956

**Table 2:** Analytical parameters obtained for the determination of Gly using the MIP-PPy/AuSPE sensor.

Analytical Parameter	MIPPy/AuSPE
Linear concentration range (ng/L)	5.0–50.0
Detection limit (ng/L)	1.6
Quantification limit (ng/L)	5.0
Sensitivity (μA/ng/L)	−3.27 × 10^−2^
Correlation coefficient (r)	0.9913

**Table 3:** Comparison of the performances of the developed sensors for Gly measurement in aqueous samples.

Type of Sensor	Linear Range	LOD	Reference
Sensors using nanomaterials for non-enzymatic indirect detection			
**AuNPs-Gr(DMF)/SPCE**	**1–50 μg/L**	**0.3 μg/L**	**This study**
**AuNPs-Gr(NAF)/SPCE**	**20–100 ng/L**	**6.6 ng/L**	**This study**
MWCNTs—IL/CuO	5 Nm–1.1 μM	1.3 nM	[22]
CPE	4.40 × 10^−8^–2.8 × 10^−6^ mol/L	2 × 10^−9^ mol/L	[61]
Cu-BTC MOF	-	1.4 × 10^−13^ mol/L	[26]
GCE/MWCNT/CuPc	0.83–9.90 μM	12.20 nM	[23]
Electrode with porous copper nano-wires	0.010–5.0 μmol/L	10.0 nmol/L	[27]
Sensors using MIP-for indirect detection			
**MIP-Ppy/AuSPE**	**5.0–50.0 ng/L**	**1.6 ng/L**	**This study**
AuSPE	5.91 nM–5.91 μM	4.73 nM	[24]
MIPPy/AuE	5–800 ng/mL	0.27 ng/mL	[62]
MIM/CL	0.024–1.04 μM	2 nM	[63]
MIP/GNPs-PGE	3.98–176.23 ng/mL	0.353 ng/mL	[64]
PPY-MIP/AuE	0.10 pM–10.0 μM	1 pM	[56]

## Data Availability

Data are contained within the article and Supplementary Materials.

## Supplementary Materials

The following supporting information can be downloaded at: https://www.mdpi.com/article/10.3390/nano14110948/s1, Figure S1: SEM images for: (a) unmodified Au electrode; (b) MIPPy before Gly removal; (c) MIPPy with specific cavities for Gly recognition; Figure S2: AC-AFM scan—Au electrode, along with roughness parameters and the histogram of local heights, determined from image analysis; Figure S3: (a) Interference studies; (b) reproducibility, (c) Repeatability and (d) time stability of MIPPy-AuSPE.
