# Supplementary material for: Electrochemical Detection of Glyphosate in Surface Water Samples Based on Modified Screen-Printed Electrodes
Source: Nanomaterials (Basel). 2024 May 28;14(11):948. doi: 10.3390/nano14110948 (PMC11173875; doi:10.3390/nano14110948)
Supplement: Supplementary file 1 [file nanomaterials-14-00948-s001.zip › nanomaterials-2964495-supplementary.pdf]

Electronic supplementary material

Electrochemical detection of glyphosate in surface water samples based on modified screen-printed electrodes

Elisabeta-Irina Geana[1*], Corina Teodora Ciucure[1], Amalia Soare[1], Stanica Enache[1], Roxana Elena Ionete, Livia Alexandra Dinu[2*]

[1] National Research and Development Institute for Cryogenics and Isotopic Technologies – ICSI Rm. Valcea, Rm. Valcea, Romania
[2] National Institute for Research and Development in Microtechnologies (IMT Bucharest), Voluntari (Ilfov), Romania
* Correspondence: irina.geana@icsi.ro; livia.dinu@imt.ro

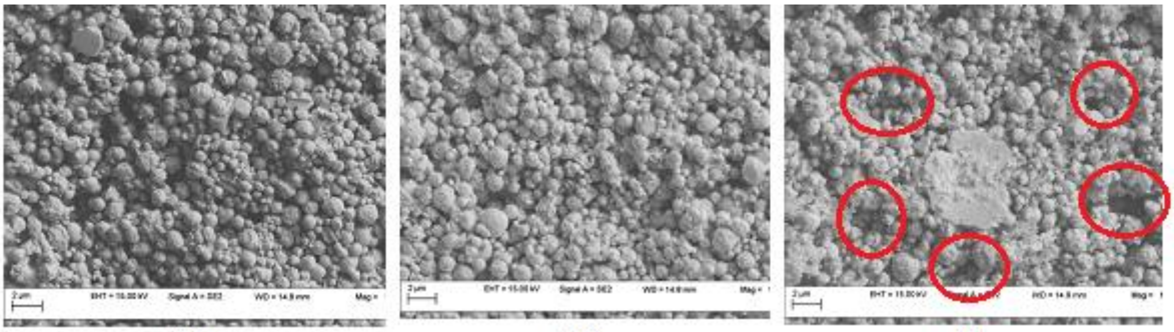

(a) (b) (c)

Figure S1. SEM images for: (a) unmodified Au electrode; (b) MIPPy before Gly removal; (c) MIPPy with specific cavities for Gly recognition.

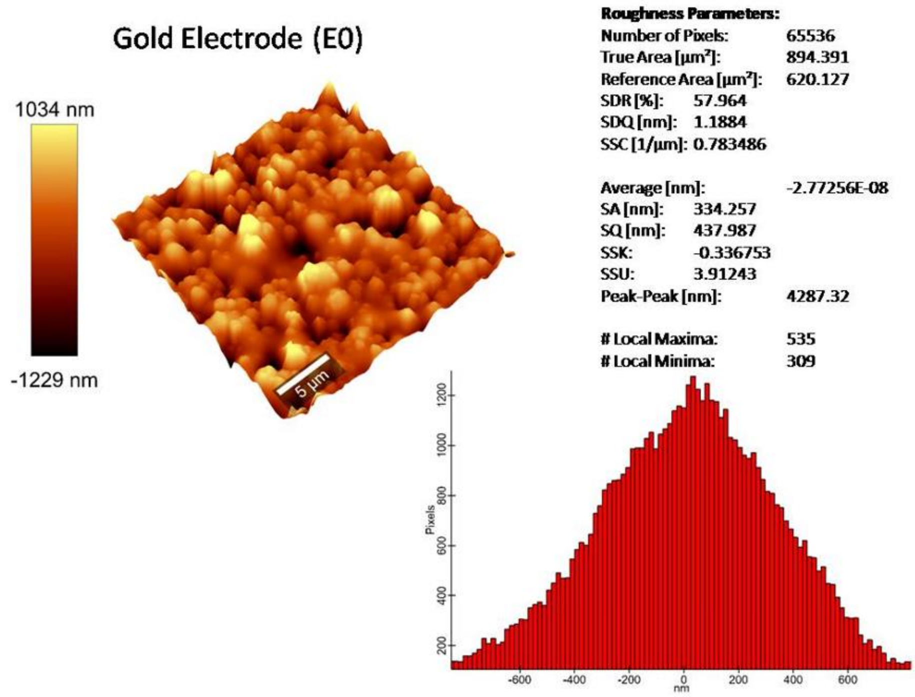

Figure S2. AC-AFM scan – Au electrode, along with roughness parameters and the histogram of local heights, determined from image analysis.

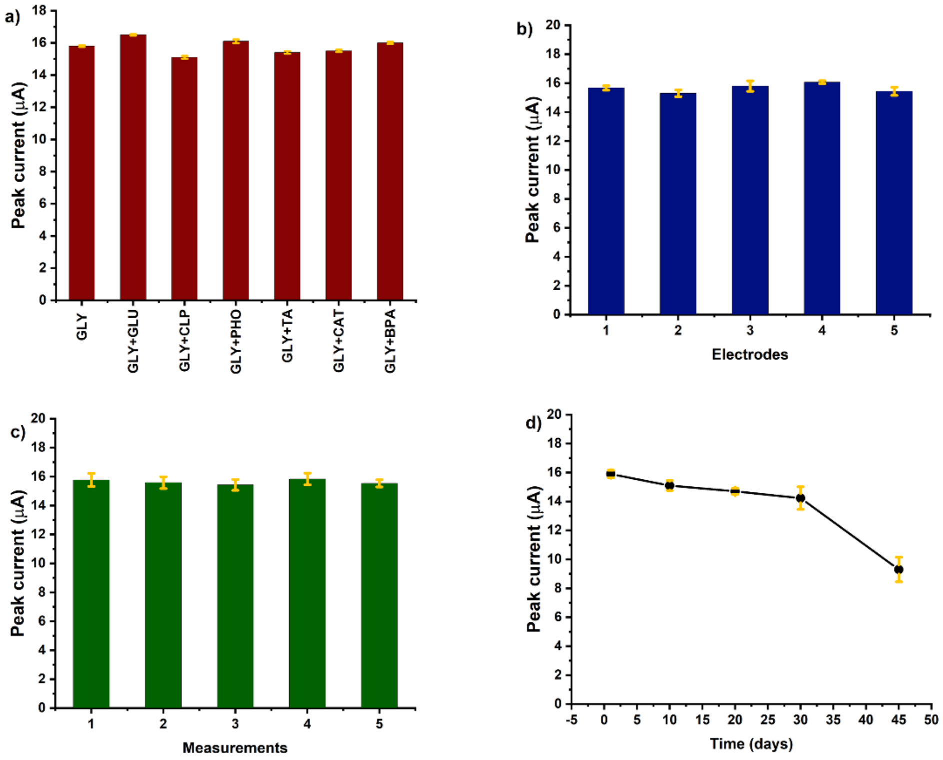

Figure S3. (a) Interference studies; (b) reproducibility, (c) Repeatability and (d) time stability of MIPPy-AuSPE.
